# Association of physical activity with adiposity in preschoolers using different clinical adiposity measures: a cross-sectional study

**DOI:** 10.1186/s12887-019-1764-4

**Published:** 2019-10-31

**Authors:** Amar Arhab, Nadine Messerli-Bürgy, Tanja H. Kakebeeke, Kerstin Stülb, Annina Zysset, Claudia S. Leeger-Aschmann, Einat A. Schmutz, Andrea H. Meyer, Simone Munsch, Susi Kriemler, Oskar G. Jenni, Jardena J. Puder

**Affiliations:** 1 Obstetric Service, Department Women-Mother-Child, Lausanne University Hospital, Lausanne, Switzerland; 2 Department of Psychology, Clinical Child Psychology and Biological Psychotherapy, University of Fribourg, Fribourg, Switzerland; 3 Child Development Center, University Children’s Hospital Zurich, Zurich, Switzerland; 4 Children’s Research Center, University Children’s Hospital Zurich, Zurich, Switzerland; 5 Epidemiology, Biostatistics and Prevention Institute, University of Zurich, Zurich, Switzerland; 6 Department of Psychology, University of Basel, Basel, Switzerland; 7 Department of Psychology, Clinical Psychology and Psychotherapy, University of Fribourg, Fribourg, Switzerland; 8 Centre Hospitalier Universitaire Vaudois (CHUV), Service of Endocrinology, Diabetes & Metabolism, Lausanne, Switzerland

**Keywords:** Body composition, Children, Body mass index, Accelerometry, SPLASHY

## Abstract

**Background:**

More research is needed about the association between physical activity (PA), sedentary behaviour (SB), and adiposity in preschoolers, particularly using more direct clinical measures of adiposity. Therefore, the main objective of this study was to investigate the association between objectively measured PA and different clinical adiposity measures in a large sample of preschoolers.

**Methods:**

Four hundred sixty-three predominantly normal-weight (77%) 2–6-year-old preschool children participated in the Swiss Preschoolers’ Health Study (SPLASHY). Physical activity was measured using accelerometers and was analyzed using 15-s (uni-axial) epoch length using validated cut-offs. Adiposity measures included body mass index (BMI), the sum of four skinfolds, and waist circumference (WC). Multilevel linear regression modeling, adjusted for age, sex and wear time, was used to assess the association between PA and SB with BMI, WC, and skinfold thickness.

**Results:**

Total PA and different PA intensities were positively and SB was inversely associated with BMI in the total sample and in the normal-weight children (*p* < 0.05). Total PA was inversely associated with skinfold thickness in overweight and obese children (*p* < 0.05), while there was only a weak association for vigorous PA (*p* > 0.05). Moderate and moderate-to-vigorous PA were positively, and SB was negatively associated with WC in the total sample and in the normal-weight children (*p* < 0.05). Additional adjustment for potential sociocultural and biological confounding variables attenuated some of the results.

**Conclusions:**

In this very young and predominantly normal-weight population, PA is positively related to BMI and WC, but this relationship is not observed in overweight and obese children. In this latter population, PA is inversely, and SB is positively related to skinfold thickness. Skinfold thickness could represent a useful and simple clinical measure of body fat in preschoolers. The role of vigorous PA in the prevention of early childhood obesity should be further investigated in future studies.

**Trial registration:**

ISRCTN ISRCTN41045021. Retrospectively registered 06 May 2014.

## Background

The recent World Health Organisation (WHO) report about early obesity prevention measures in young children recommends the promotion of physical activity (PA) [1]. In school-aged children and adolescents, higher levels of PA have been shown to be protective against adiposity [2–4]. However, the association between PA and adiposity in preschool children seems to depend on the outcome measure of adiposity. Indeed, most studies that have used body mass index (BMI) to assess adiposity failed to find an association, whereas studies that used percent bodyfat (BF) as outcome measure for adiposity found evidence for an inverse association [5]. Furthermore, results for sedentary behaviour (SB) and adiposity are somewhat inconsistent [6–8] with null associations for objectively assessed SB [6], and a positive association of SB with waist circumference (WC) in girls [7]. Therefore, more research is needed to understand the association between PA, SB and adiposity in preschool children.

The use of practice-based and inexpensive clinical measures of adiposity such as BMI, skinfold thickness, and WC is primordial for utilization in larger studies and clinical practice. Most studies have assessed the association between objectively measured PA and BMI, but a few have looked at WC and skinfold thickness. Using BMI as a proxy od adiposity, the majority of studies found no associations between PA and BMI in preschoolers [9–17]. However, some studies found an inverse association [18–20] whereas others found a positive association [7, 21, 22] between these two parameters. To the best of our knowledge, four studies investigated the association between PA and WC in preschool children [7, 17, 23, 24]. Two studies found no associations [17, 24], and one found an inverse relationship between moderate-to-vigorous PA and WC [23]. The fourth study found an inverse association between moderate-to-vigorous PA and WC, but only among a subgroup of obese girls (with a WC of ≥90th percentile) [7]. Three studies have investigated the association of objectively measured PA with skinfold thickness in preschool children. They showed either no association [24], or that higher levels of PA were associated with reduced skinfold thickness [23, 25]. However, many studies in preschoolers have used more complex and expensive measures like dual-energy X-ray absorptiometry (DXA) or air-displacement plethysmography (ADP) when assessing the association of PA with body fat [6, 8, 15, 17, 26]. Nevertheless, these new technologies are not always suitable in large studies. Thus, using simple and inexpensive clinical measures of total or central body fat such as skinfold thickness and WC would allow for larger community-based studies in preschool children and thus increase external validity.

It has been suggested that high-intensity PA improves body composition and prevents obesity in children, possibly by reducing stem cell differentiation into fat mass [27]. In accordance with this observation, five studies using DXA or ADP have found an inverse association between vigorous PA and body fat [6, 8, 15, 17, 26]. In addition, young children’s PA is highly intermittent, and is characterized by brief and sporadic bursts of energy [28]. Therefore, the use of short epoch lengths of 15-s intervals of accelerometer recordings have been suggested, especially for higher intensities [29, 30].

The main aim of this study was to determine the cross-sectional associations between physical activity with adiposity in a sample of 2–6-year-old preschoolers using different practice-based clinical adiposity measures: BMI, skinfold thickness and WC. We were further interested in evaluating the impact of different PA intensities and SB on adiposity.

## Methods

### Study sample and design

The Swiss Preschoolers’ Health Study (SPLASHY) is a multi-site prospective cohort study including 476 preschool children within two sociocultural areas of Switzerland (German and French speaking part) (ISRCTN41045021). Preschool children were recruited from 84 childcare centers within five cantons (provinces) of Switzerland (Aargau, Bern, Fribourg, Vaud, Zurich), which together made up 50% of the Swiss population. Recruitment started between November 2013 and October 2014 when children were 2–6 years old. The detailed study design and the overall objectives have been previously described [31]. For the present analysis, children with no BMI data (*n* = 13) were excluded. Thus, the final analysis included 463 children. The study was approved by all local ethical committees (No 338/13 for the Ethical Committee of the Canton of Vaud as the main ethical committee). Parents provided written informed consent. This current analysis focuses on the baseline cross-sectional data.

### Assessment of PA

PA was objectively monitored using an accelerometer (w/GT3X-BT, ActiGraph, Pensacola, FL, USA). Children were asked to continuously wear the accelerometer around the hip for five weekdays and two weekend days including the nights. The device was removed for water-based activities, e.g. showing or swimming. Children, parents, and childcare staff received detailed instructions on the use of the activity monitor. Physical activity data (raw data) were collected at a sampling frequency of 30 Hz, downloaded in 3-s epochs. For data analysis, periods of 20 min and more of consecutives zero readings were removed and considered as non-wear time. All recordings between 9 pm and 7 am were excluded as this most likely reflected the hours spent sleeping. A minimum of 10 h of recording per day was required for inclusion in analysis. In a first analysis, we analyzed all children with at least one valid day. In a second step, we only analyzed children with at least two valid weekdays and one valid weekend day and compared them with children with one valid day of accelerometer.

Physical activity data was sampled at a frequency of 30 Hz, downloaded in three-seconds epochs and aggregated to 15-s epoch length. Moderate-to-vigorous PA was defined as ≥420 counts/15-s by Pate [32] and SB was defined as ≤25 counts/15-s by Evenson [33]. The Pate et al. accelerometer cut-points are the only ones validated for 15-s epoch in preschool children and these cut-points are validated for the uni-axial vector model only (vertical axis) [32]. For their validation, they used an indirect method of a portable metabolic system with a mask. Similarly, Evenson et al. used a uniaxial accelerometer to validate SB cut-points using a portable metabolic system with a mask [33]. Data collection and processing criteria were based on a recent review [34].

### Assessment of adiposity

The 3 different adiposity measures (BMI, skinfold thickness, and WC) were performed in the childcare center by 5 different well-trained examiners (CL, AZ, KS, AA, NM). Height was measured to the nearest 0.1 cm with a stadiometer and weight to the nearest 0.1 kg (Seca, Basel, Switzerland) using standardized procedures. BMI was then calculated as weight/height squared (kg/m^2^). All measurements were taken barefoot and in light clothing. Age and gender adjusted BMI percentiles were calculated and overweight/obese (OW/OB) were defined based on the WHO criteria [35]. Skinfold thickness was measured using standard procedures [36] in triplicate to the nearest 0.1 mm with Harpenden calipers (HSK-BI, British Indicators, UK) at the triceps, biceps, subscapular, and suprailiac crest. The sum of all four skinfolds was calculated and referred to as “skinfold thickness or body fat”. Waist circumference was measured in duplicate without clothing midway between the iliac crest and the lowest border of the rib cage to the nearest 0.1 cm with a flexible tape.

### Potential confounding variables

Potential confounding variables known to be related to childhood obesity and PA were included. Socioeconomic status (SES) was calculated by coding the occupational status of both parents using the International Socio-Economic Index (ISEI) value [37]. The maximal SES was then determined by the selection of the highest of the parental ISEI values. Sociocultural regions were defined by language and geographical region (German speaking, versus French speaking, part of Switzerland) [38]. Parents filled out a general health questionnaire reporting the maternal weight and height which were used to calculate maternal BMI.

### Statistical analysis

Descriptive statistics were calculated using means ±SD for continuous variables, or percentages for categorical variables. All outcome variables were checked for normal distribution and only vigorous PA was log-transformed.

Multilevel linear regression modeling including childcare as a random factor was used to assess the association between PA and adiposity. Predictors were total PA, time spent in the different PA intensities, and in SB. Each predictor was tested in a separate model. Outcomes were the three different measures of body composition. The model contained a random intercept for each childcare center.

We also tested if these associations were different between children who did have at least two valid weekdays and one valid weekend day (*n* = 392: 92% of all children who had BMI data) and the children with at least one valid day (*n* = 33: 8% of children who had BMI data) of PA accelerometer measurements. We did this by adding a dummy variable regarding these 3 days validity criteria to the model together with its interaction with the predictor PA. This allowed us to test whether having one valid day of PA measurements led to different estimated coefficients for PA compared to three valid days. However, interaction terms for PA measures were not significant across all three body composition outcomes (all *p* > 0.05) and estimates were always comparable between the two groups. In addition, groups did not differ in mean values of PA and body composition, except for higher WC and SB in the children with three valid days of PA data (*p* < 0.05). We henceforth only report the results of the entire sample of children having at least one valid day of accelerometer measurements. Missing data were not imputated.

In accordance with most previous studies, analyses testing the associations between PA and adiposity were adjusted for the children’s age, sex and wear time and represented the basic model. In a further step, we additionally adjusted for sociocultural (ISEI max, sociocultural region) and biological (maternal BMI) confounders. Consequently, the sample size of the second model was reduced due to fewer available data for confounding variables (ISEI max and maternal BMI).

As the fat-mass and fat-free mass content differ between preschoolers with normal-weight (NW) and preschoolers with OW/OB, the relationship between PA and adiposity measures were also assessed in subgroup analyses according to weight status.

Analyses were performed using 15-s epoch length using Pate cut-points [32]. For the ease of interpretation, regression coefficients from all models were converted: coefficients were modified to represent the average unit change in the outcome per unit increase in the independent variable by simply multiplying it with 100 for total PA (per 100 counts per minute) and by 10 for the time spent in different PA and sedentary intensities (per 10 min). Statistical significance was set at *P* < 0.05 for all analyses.

## Results

The descriptive characteristics for all children are summarized in Table 1. Mean age was 3.9 ± 0.7 years and 47% of the participating children were girls. Overweight and obesity prevalence rates were 18 and 5% according to WHO criteria [35]. All prevalence rates were similar between girls and boys (*p* > 0.05).

**Table 1:** Descriptive characteristics of the participants stratified by weight status

Characteristics	Total (*N* = 463)		NW (*N* = 357)		OW/OB (*N* = 106)		*P*-value
	n (% missing)	Value	n (% missing)	Value	n (% missing)	Value	
Age (years)	463	3.9 ± 0.7	357	3.9 ± 0.7	106	3.8 ± 0.8	0.13
Gender	463		357		106		
Boys, n (%)		246 (53)		190 (53)		56 (53)	0.94
Girls, n (%)		217 (47)		167 (47)		50 (47)	
Socioeconomic/cultural							
ISEI max.	424 (8)	61.8 ± 16.1	329 (8)	61.8 ± 15.7	95 (10)	61.8 ± 17.4	0.73
Sociocultural region	463		357		106		
French speaking, n (%)		121 (26)		96 (27)		25 (24)	0.45
German speaking, n (%)		342 (74)		261 (73)		81 (76)	
Biological							
Maternal BMI (kg/m^2^)	434 (6)	22.7 ± 3.8	337 (6)	22.5 ± 3.7	97 (8)	23.5 ± 4.2	**0.02**
Body composition							
Body mass index (kg/m^2^)	463	16.0 ± 1.4	357	15.5 ± 0.9	106	17.8 ± 1.1	**< 0.001**
Waist circumference (cm)	454 (2)	51.9 ± 3.5	350 (2)	50.9 ± 3.0	104 (2)	55.1 ± 3.4	**< 0.001**
Skinfold thickness (mm)	425 (9)	25.9 ± 5.5	328 (8)	24.8 ± 4.6	104 (2)	29.8 ± 6.5	**< 0.001**
Physical activity							
TPA (cpm)	425 (8)	624 ± 155	321 (10)	620 ± 146	104 (2)	636 ± 176	0.51
LPA (min/d)	425 (8)	300 ± 34	321 (10)	299 ± 34	104 (2)	301 ± 33	0.56
MPA (min/d)	425 (8)	70 ± 20	321 (10)	69 ± 19	104 (2)	71 ± 21	0.33
MVPA (min/d)	425 (8)	92 ± 30	321 (10)	91 ± 29	104 (2)	95 ± 34	0.39
VPA (min/d)	424 (8)	23 ± 12	320 (10)	22 ± 11	104 (2)	24 ± 15	0.85
SB (min/d)	425 (8)	372 ± 50	321 (10)	374 ± 50	104 (2)	365 ± 53	0.12

Total population is based on children with valid BMI data

*NW* normal-weight, *OW/OB* overweight/obese, *ISEI* international socio-economic index, *BMI* body mass index, *TPA* total physical activity, *LPA* light physical activity, *MPA* moderate physical activity, *MVPA* moderate-to-vigorous physical activity, *VPA* vigorous physical activity, *SB* sedentary behaviour, *cpm* counts per minute, *min/d* minutes per day

Cut-points for the physical activity intensities are based on Pate [32]

Differences in baseline characteristics between NW and OW/OB were calculated using the WHO criteria [35]

Data in bold signifies *p* < 0.05

Age, sex and wear time adjusted associations between PA and SB with BMI, skinfold thickness, and WC are presented in Table 2a. In the total sample, total PA and time spent in light, moderate, and moderate-to-vigorous PA were positively associated with BMI (*p* < 0.05). For example, increasing total PA by 100 cpm/day or the time spent in moderate PA by 10 min/day were both associated with a 0.1 kg/m^2^ higher BMI. Sedentary behaviour showed an inverse relation with BMI (*p* < 0.05). In the NW children, total PA and all PA intensities were positively associated and SB was inversely associated with BMI (*p* < 0.05), while there were no associations in the OW/OB children.

**Table 2:** Associations between physical activity, sedentary behaviour, and adiposity

a) Adjusted for age, sex and wear time						
	BMI					
	Total (*n* = 425)		NW (*n* = 321)		OW/OB (*n* = 104)	
	β-coefficient (95% CI)	*P*-value	β-coefficient (95% CI)	*P*-value	β-coefficient (95% CI)	*P*-value
TPA (cpm)	0.10 (0.02, 0.20)	**.02**	0.09 (0.02, 0.15)	**.01**	− 0.001 (− 0.14, 0.14)	.98
LPA (min/d)	0.05 (0.01, 0.10)	**.001**	0.05 (0.02, 0.08)	**.001**	0.01 (− 0.06, 0.08)	.80
MPA (min/d)	0.10 (0.05, 0.20)	**.001**	0.10 (0.05, 0.15)	**.001**	0.04 (− 0.08, 0.17)	.50
MVPA (min/d)	0.08 (0.03, 0.10)	**.002**	0.06 (0.02, 0.09)	**.001**	0.01 (− 0.06, 0.09)	.71
^b^VPA (min/d)	2.20 (− 0.51, 4.83)	.11	2.08 (0.11, 4.05)	**.04**	−1.08 (−5.43, 3.28)	.62
SB (min/d)	−0.05 (− 0.08, − 0.02)	**.001**	− 0.04 (− 0.06, − 0.02)	**.001**	− 0.01 (− 0.05, 0.04)	.71
	Skinfold thickness					
	Total (*n* = 395)		NW (*n* = 300)		OW/OB (*n* = 95)	
	β-coefficient (95% CI)	*P*-value	β-coefficient (95% CI)	*P*-value	β-coefficient (95% CI)	*P*-value
TPA (cpm)	−0.16 (− 0.51, 0.19)	.37	0.05 (− 0.30, 0.39)	.80	− 0.79 (−1.52, − 0.06)	**.04**
LPA (min/d)	0.07 (− 0.09, 0.23)	.40	0.11 (− 0.05, 0.26)	.19	− 0.26 (− 0.64, 0.13)	.19
MPA (min/d)	0.09 (− 0.19, 0.38)	.52	0.18 (−0.09, 0.46)	.19	−0.42 (−1.09, 0.25)	.21
MVPA (min/d)	−0.03 (− 0.22, 0.17)	.79	0.04 (− 0.15, 0.23)	.66	− 0.33 (− 0.75, 0.09)	.12
^b^VPA (min/d)	−7.22 (−17.82, −3.39)	.18	−1.77 (− 12.10, 8.57)	.74	−22.97 (−46.54, 0.60)	.06
SB (min/d)	− 0.03 (− 0.14,0.08)	.59	− 0.07 (− 0.17, 0.04)	.20	0.21 (− 0.04, 0.46)	.10
	Waist circumference					
	Total (*n* = 416)		NW (*n* = 314)		OW/OB (*n* = 102)	
	β-coefficient (95% CI)	*P*-value	β-coefficient (95% CI)	*P*-value	β-coefficient (95% CI)	*P*-value
TPA (cpm)	0.16 (−0.07, 0.39)	.16	0.17 (− 0.05, 0.39)	.13	0.02 (− 0.38, 0.42)	.92
LPA (min/d)	0.05 (− 0.06, 0.15)	.38	0.07 (− 0.03, 0.16)	.20	−0.10 (− 0.30, 0.10)	.34
MPA (min/d)	0.26 (0.07, 0.44)	**.006**	0.25 (0.08, 0.42)	**.004**	0.05 (−0.31, 0.41)	.79
MVPA (min/d)	0.15 (0.03, 0.27)	**.02**	0.13 (0.02, 0.25)	**.03**	0.06 (−0.02, 0.29)	.57
^b^VPA (min/d)	4.51 (−2.27, 11.28)	.19	4.65 (−1.75, 11.06)	.10	2.67 (−10.03, 15.36)	.68
SB (min/d)	−0.07 (−0.14, − 0.004)	**.04**	−0.08 (− 0.14, 0.01)	**.02**	0.01 (− 0.12, 0.14)	.87
b) Adjusted for age, sex, wear time, sociocultural and biological confounding variables^a^						
	BMI					
	Total (*n* = 391)		NW (*n* = 298)		OW/OB (*n* = 93)	
	β-coefficient (95% CI)	*P*-value	β-coefficient (95% CI)	*P*-value	β-coefficient (95% CI)	*P*-value
TPA (cpm)	0.07 (−0.03, 0.16)	.16	0.07 (0.001, 0.14)	**.048**	−0.005 (− 0.16, 0.15)	.94
LPA (min/d)	0.03 (−0.01, 0.07)	.21	0.04 (0.01, 0.07)	**.02**	−0.01 (− 0.09, 0.06)	.76
MPA (min/d)	0.11 (0.02, 0.17)	**.01**	0.09 (0.03, 0.14)	**.002**	0.03 (−0.10, 0.16)	.65
MVPA (min/d)	0.06 (0.01, 0.11)	**.03**	0.05 (0.01, 0.09)	**.01**	0.01 (−0.07, 0.01)	.75
^b^VPA (min/d)	1.30 (−1.44, 4.11)	.34	1.66 (−0.44, 2.57)	.12	−0.90 (−5.40, 3.59)	.69
SB (min/d)	−0.03 (− 0.06, − 0.003)	**.03**	−0.04 (− 0.06, − 0.01)	**.001**	−0.0002 (− 0.05, 0.05)	.99
	Skinfold thickness					
	Total (*n* = 362)		NW (*n* = 277)		OW/OB (*n* = 85)	
	β-coefficient (95% CI)	*P*-value	β-coefficient (95% CI)	*P*-value	β-coefficient (95% CI)	*P*-value
TPA (cpm)	−0.18 (−0.57, 0.20)	.35	0.06 (−0.32, 0.44)	.75	−0.57 (−1.36, 0.22)	.15
LPA (min/d)	0.06 (−0.11, 0.24)	.49	0.16 (−0.01, 0.32)	.07	−0.32 (− 0.69, 0.06)	.10
MPA (min/d)	0.07 (−0.24, 0.39)	.65	0.20 (−0.09, 0.51)	.18	−0.34 (−1.01, 0.33)	.32
MVPA (min/d)	−0.04 (− 0.25, 0.17)	.69	0.04 (− 0.16, 0.25)	.69	− 0.18 (− 0.61, 0.26)	.42
^b^VPA (min/d)	−9.67 (−21.22, 1.89)	.10	−3.55 (−14.92, 7.82)	.54	−16.28 (−40.19, −7.63)	.18
SB (min/d)	−0.03 (− 0.15, 0.10)	.68	−0.09 (− 0.21, 0.02)	.10	0.18 (− 0.07 0.44)	.16
	Waist circumference					
	Total (*n* = 386)		NW (*n* = 294)		OW/OB (*n* = 92)	
	β-coefficient (95% CI)	*P*-value	β-coefficient (95% CI)	*P*-value	β-coefficient (95% CI)	*P*-value
TPA (cpm)	0.09 (−0.14, 0.34)	.43	0.14 (− 0.09, 0.37)	.22	0.07 (− 0.40, 0.53)	.77
LPA (min/d)	0.03 (−0.08, 0.14)	.61	0.05 (−0.05, 0.16)	.30	−0.07 (− 0.29, 0.15)	.55
MPA (min/d)	0.22 (0.02, 0.41)	**.03**	0.23 (0.05, 0.41)	**.01**	0.08 (−0.31, 0.47)	.68
MVPA (min/d)	0.10 (−0.02, 0.25)	.08	0.11 (−0.01, 0.24)	.07	0.09 (−0.16, 0.34)	.47
^b^VPA (min/d)	1.74 (−5.42, 8.91)	.63	2.85 (−3.99, 9.69)	.41	2.49 (−11.08, 16.06)	.72
SB (min/d)	−0.06 (−0.13, 0.02)	.14	−0.07 (− 0.14, 0.003)	.06	−0.01 (− 0.16, 0.13)	.84

Data are shown using 15-s epoch uniaxial vector

^a^Analyses are adjusted for age, sex, wear time, International Socio-Economic Index (ISEI), socio-cultural region, and maternal BMI

Data are shown per changes in 100 cpm and per 10 min intervals for the time in the respective intensities

*NW* normal-weight, *OW/OB* overweight/obese, *CI* confidence interval, *TPA* total physical activity, *LPA* light physical activity,*MPA* moderate physical activity, *MVPA* moderate-to-vigorous physical activity, *VPA* vigorous physical activity, *SB* sedentary behaviour, *cpm* counts per minute, *min/d* minutes per day

Weight status, NW and OW/OB, were defined using the WHO criteria [35]

^b^values are log-transformed

Data in bold signifies *p* < 0.05

There were no significant associations between the different PA measures and skinfold thickness in the total sample or in the NW children. However, in the OW/OB children, total PA was inversely associated with skinfold thickness (*p* < 0.05) and this inverse relationship was weak for vigorous PA (*p* > 0.05).

Moderate and moderate-to-vigorous PA were positively associated with WC in the total population and in the NW children (*p* < 0.05). Sedentary behaviour was inversely associated with WC in the total population and in the NW children (p < 0.05). Similar to BMI, PA was not related to WC in the OW/OB children.

The associations between PA and adiposity after further adjustments for sociocultural and biological confounders are presented in Table 2b and show a slightly reduced sample size. The following associations remain significant after these adjustments: Moderate and moderate-to-vigorous PA remained positively associated with BMI in the total sample and total PA and all intensities except for vigorous PA remained significant in the NW children (*p* < 0.05). Also, SB remained negatively associated with BMI in the total population and in the NW children (*p* < 0.05). Regarding WC, moderate PA remained associated with WC in the total sample (*p* < 0.05) and in the NW children (*p* < 0.05).

## Discussion

In this large and very young sample of predominantly NW preschoolers, we found that PA was positively related to BMI and to WC. This association was only observed in the NW, but not in the OW/OB children. Total and vigorous PA were inversely associated with skinfold thickness in the OW/OB children. Based on our results, we may conclude that BMI and WC are not an ideal measure of excess body fat in young and healthy preschoolers. Especially, BMI might rather be an indicator of fat-free mass in this population. On the other hand, skinfold thickness may represent a useful, valid and inexpensive research and clinical measure of body fat in this population*.*

Most previous studies using BMI to express adiposity in preschoolers found no significant association between objectively measured PA and BMI and/or BMI z-score [9–15, 17]. Since in the included studies most of the children were NW or overweight rather than obese, it’s possible that misclassification for none or mild excess adiposity obscured the association between PA and BMI. Three studies found an inverse association with BMI [18–20]. The reported differences between weight groups were often driven by very obese children at the “extreme” spectrum. Similar to our results, 3 studies found a positive association between PA and BMI [7, 21, 22]. For example, Espana-Romero et al. [7] reported a significant positive association between moderate-to-vigorous PA and BMI z-score in boys only with a BMI up to the 50th percentile. The authors suggested that the positive association maybe explained by greater fat-free mass among more active boys. Likewise, the positive association between PA and BMI in our study was only found in the NW children. In these NW children, BMI accuracy to measure adiposity is indeed limited and BMI differences are largely driven by differences in fat-free mass [39]. A study describing the relation of fat-free mass and fat mass to BMI, showed that in NW 5–8-year-old children fat-free mass was a stronger predictor of BMI than fat mass [40]. Thus, BMI reliability is uncertain for this age group to define adiposity, especially in the non-obese.

We found that PA was negatively associated with skinfold thickness. These associations were observed in the OW/OB children. Similarly, several studies using DXA or ADP found an inverse association between vigorous PA with body fat [6, 8, 15, 17, 26]. However, it is not always suitable to use DXA or ADP in large epidemiological studies because of cost, irradiation exposure, and limited availability outside research settings [41]. Alternative methods such as skinfold thickness are more practical, especially as they highly correlate with DXA in children (r = 0.90) [42]. Similarly, two previous studies found an inverse association between objectively measured PA and skinfold thickness in preschool children [23, 25]. Skinfold thickness could thus represent a very useful and simple measure of body fat in community-based studies and clinical practice involving preschoolers.

The particular importance of vigorous PA has been shown in previous studies using DXA. In a large cohort of British preschoolers, vigorous PA, but not other PA measures, was inversely associated with total and abdominal adiposity [6]. Similarly, several studies showed that children with higher vigorous PA had either lower body fat or were less likely to gain body fat compared to children with low vigorous PA levels [8, 20, 26]. Our results showed similar trend using skinfold measures. Therefore, the role of vigorous PA in the prevention of early childhood obesity should be further investigated in future studies to bring more evidence.

As visceral adiposity is related to numerous cardiovascular and metabolic risk factors [43], we evaluated the use of WC as a potential measure of central body fat. We found a positive association between moderate and moderate-to-vigorous PA with WC and a negative association between SB and WC in the total population and in the NW children, while no association was found in the OW/OB children. To our knowledge, four previous studies investigated this association in preschoolers, but children in three of these studies had higher WC measures compared to our sample. In contrast to our results, Leppännen et al. [17] and Metcalf et al. [24] found no association between PA and WC, noting that the former used wrist placement to capture PA. A third study was done in 2-year old toddlers, and found that moderate-to-vigorous was inversely associated with WC [23]. The fourth study did not find an association between PA and WC in the total sample. However, among a subgroup of obese girls (≥90th WC percentile), they found a positive association between SB and WC and a negative association between moderate-to-vigorous PA and WC [7]. These discrepancies in results could be explained by differences in WC or prevalence of OW/OB between studies and/or by differences in the age of the children as the shape of the children and the WC changes in toddlers. The overall results of our data and the previous studies could suggest that WC might be an interesting and distinctive measure of visceral obesity in preschoolers, but only in the presence of obesity. Furthermore, it is often difficult to reliably measure WC in young preschoolers because of frequent small changes in posture, abdominal inflation or deflation, low abdominal musculature, and as they get easily tickled. This might even be more difficult in toddlers.

### Strength and limitations

Strengths of the current study included the relatively large sample size from regions that are representative of Switzerland. We also investigate a very young population that is younger than most cited studies which enables us to examine the association between health indicators early in the developmental process when prevalence of pathology is still low. The inclusion of different valid and inexpensive clinical measures of adiposity represent further strengths of this trial. However, the cross-sectional design of the study precludes our ability to infer a causal relation between PA and adiposity in preschoolers. Performing multiple comparison could potentially lead to biases and is thus a limitation of our study. Also, our accelerometry data processing method to identify and remove sleep might have affected estimates of PA and SB. Indeed, it has been shown that different Actigraph scoring rules to identify and remove periods of sleep affected estimates of PA and sedentary time in children [44]. Furthermore, the short duration of PA spent in vigorous physical activity intensity might confound certain results. However, the duration of vigorous PA in the current study is very similar to other studies in preschoolers [6]. Finally, similar to previous trials, our study is limited by lack of data on dietary intake, which may have also affected the associations observed,

## Conclusions

In conclusion, we observed that in predominantly NW preschoolers PA was positively related to BMI and WC. This association was only observed in the NW, but not in the OW/OB children. Physical activity, particularly total and vigorous PA, were inversely related to skinfold thickness in the OW/OB children. BMI and WC alone do not adequately reflect adiposity in this population, and BMI is possibly even an indicator of fat-free mass in healthy NW preschool children. Therefore, skinfold thickness or other direct measures of body fat are needed for research and clinical practice. Also, more evidence is needed to understand the role of vigorous PA in the prevention of obesity in the early years. Prospective study designs are needed to further examine the association between PA and body composition in young children and to define the best measures to assess both PA and adiposity.

## Data Availability

The datasets used and/or analyzed during the current study are available from the corresponding author on a validated request.

## Abbreviations

ADP: Air-displacement plethysmography
BMI: Body mass index
DXA: Dual-energy x-ray absorptiometry
IOTF: International obesity task force
ISEI: International socio-economic index
NW: Normal-weight
OW/OB: Overweight/obese
PA: Physical activity
SB: Sedentary behaviour
SES: Socioeconomic status
WC: Waist circumference
WHO: World health organisation
